# BHDPC Is a Novel Neuroprotectant That Provides Anti-neuroinflammatory and Neuroprotective Effects by Inactivating NF-κB and Activating PKA/CREB

**DOI:** 10.3389/fphar.2018.00614

**Published:** 2018-06-25

**Authors:** Chuwen Li, Tongkai Chen, Hefeng Zhou, Yu Feng, Maggie P. M. Hoi, Dan Ma, Chao Zhao, Ying Zheng, Simon M. Y. Lee

**Affiliations:** ^1^State Key Laboratory of Quality Research in Chinese Medicine, Institute of Chinese Medical Sciences, University of Macau, Macau, China; ^2^Institute of Clinical Pharmacology, Guangzhou University of Chinese Medicine, Guangzhou, China; ^3^Department of Clinical Neurosciences, Wellcome Trust-MRC Cambridge Stem Cell Institute, University of Cambridge, Cambridge, United Kingdom

**Keywords:** BHDPC, neuroinflammation, microglia, NF-κB, CREB, PKA

## Abstract

Microglia-mediated neuroinflammatory responses are inevitable and important pathological processes in several kinds of disorder of the central nervous system (CNS). Therefore, alleviating activated microglia-induced inflammatory process might be a valuable therapeutic approach to neuroinflammation-related diseases. In the present study, we investigated BHDPC, a novel neuroprotectant discovered in our previous study that had anti-inflammatory effects under neuroinflammatory conditions. First, we found that BHDPC could inhibit neuroinflammatory responses and promote microglial M2 phenotype polarization in both lipopolysaccharide (LPS)-activated BV-2 microglia l cells. Furthermore, BHDPC provided protective actions against neuroinflammation-induced neurotoxicity in HT22 mouse hippocampal cells co-cultured with activated BV-2 microglia. Further experiments demonstrated that BHDPC could suppress LPS-induced activation of transcription factor nuclear factor kappa B (NF-κB) via interfering with the degradation of the inhibitor of kappa B (IκB) and phosphorylation of IκB, the IκB kinase (IKK). Moreover, we also found that BHDPC could induce phosphorylation of cAMP-dependent protein kinase A (PKA) and cAMP-response element-binding protein (CREB) in BV-2 microglial cells. Also, using the PKA-specific inhibitor, we found that BHDPC-induced CREB phosphorylation was dependent on PKA, which also contributed to BHDPC-mediated anti-inflammation and neuroprotection.

## Introduction

The inflammatory response is common to the pathological processes of various kinds of damage and disorders of the CNS (Xanthos and Sandkuhler, 2014). Microglia are immune-surveillance cells that also serve as major components of inflammatory responses (Mietto et al., 2015). In response to various microenvironmental disturbances, microglia can be phenotypically polarized into a classical (pro-inflammatory; M1) or an alternative (anti-inflammatory; M2) phenotype (David and Kroner, 2011; Saijo and Glass, 2011; Joseph and Venero, 2013; Mantovani et al., 2013). Generally, activated microglial cells can mediate neuroinflammatory responses via generating pro-inflammatory cytokines, including TNF-α, IL-1β, and IL-6, and the up-regulation of iNOS/NO and prostaglandin E_2_ (PGE_2_)/COX-2 pathways, whereas M2 phenotype microglia have been demonstrated to be associated with the expression of anti-inflammatory M2 markers including arginase-1 (Arg-1) and mannose receptor (CD206). Functionally, the M1 phenotype microglia could induce or exacerbate cerebral lesions and neuronal degeneration. In contrast, the M2 microglia confers neuroprotection and promotes recovery and remodeling (Lu et al., 2010; Zhou et al., 2014b). Therefore, pharmacological inhibition of aberrant microglial activation and/or promotion of microglial cells toward the M2 stage might be a potential regimen for treating neuroinflammatory and neurodegenerative diseases.

The activated microglia-mediated neuroinflammatory responses are characterized by pro-inflammatory molecule production, which is well known to be regulated by the transcription factor NF-κB signaling pathway (Baeuerle and Henkel, 1994; Karin and Ben-Neriah, 2000). Normally, inactive NF-κB proteins are bound to the IκB proteins and sequestered in the cytosol. To induce NF-κB activation, the IKK firstly phosphorylates and degrades IκB, which dissociates NF-κB from complex and induces NF-κB translocation into the nucleus. Once in the nucleus, NF-κB can interact with the transcriptional coactivator CREB-binding protein (CBP), further triggering transcription of various pro-inflammatory genes including cytokines and their receptors. Activation of NF-κB can also involve phosphorylation of several residues in the transactivation domain of p65 subunit, among which Ser536 is well characterized (Baeuerle and Henkel, 1994; Karin and Ben-Neriah, 2000). Suppression of NF-κB activation is an action of many reported anti-inflammatory agents (Yuan et al., 2016; de Oliveira et al., 2018).

cAMP-response element-binding protein is a cellular transcription factor and is one of the best understood phosphorylation-dependent transcription factors (Lonze et al., 2002; Holz et al., 2006; Nguyen et al., 2007). CREB mediates genes closely associated with neuronal survival, neural differentiation, and neurite outgrowth (Lonze et al., 2002; Holz et al., 2006; Nguyen et al., 2007). Several kinases have been shown to promote phosphorylation of CREB at its transcription-activating site, including the PKA, protein kinase C, calmodulin kinases and pp90 ribosomal S6 kinase (Medzhitov and Horng, 2009; Ghosh et al., 2016; Park et al., 2016). Phosphorylation of CREB promotes CREB binding to the transcriptional coactivator CBP and formation of CREB-CBP complex, which could suppress NF-κB-CBP complex formulation and further block the transcription of inflammatory genes (Medzhitov and Horng, 2009; Ghosh et al., 2016; Park et al., 2016). Moreover, accumulating evidence also demonstrates that the CREB-CBP complex may also induce gene expression of anti-inflammatory cytokines in activated microglia, which are typically involved in microglia de-activation and microglia polarization into the M2 phonotype (Ajmone-Cat et al., 2003; Ghosh et al., 2016). On the other hand, activation of the PKA/CREB pathway could enhance the expression of detoxifying and antioxidant enzymes, which could protect microglial cells and neighboring neurons from inflammation-mediated damage (Ulbrich et al., 2017).

**FIGURE 1:**
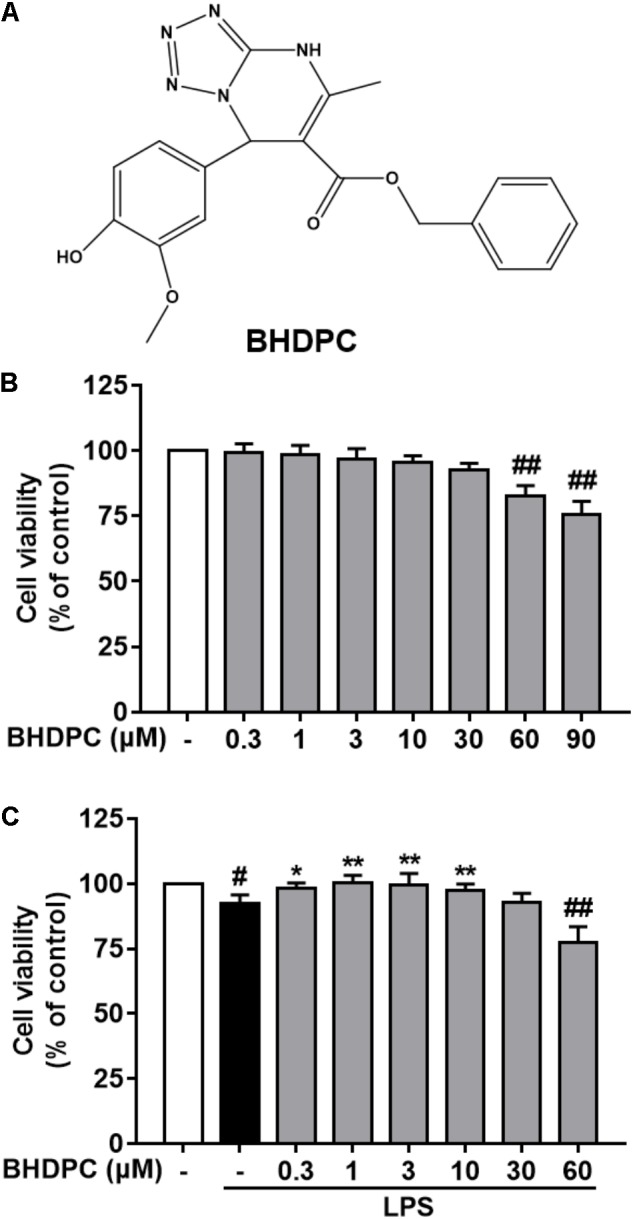
**(A)** Chemical structure of BHDPC and its effects on the cell viability of BV-2 microglial cells. BV-2 microglial cells were treated with BHDPC with or without incubation of lipopolysaccharide (LPS) (500 ng/mL) for for 24 h. **(B)** Effects of BHDPC alone on the cell viability. **(C)** Effects of BHDPC on the cell viability with LPS stimulation. Data are presented as means ± SEM of three independent experiments in triplicate. Control group was untreated cells. ^#^*P <* 0.05 and ^##^*P <* 0.01, versus control group; ^∗^*P <* 0.05 and ^∗∗^*P <* 0.01, versus LPS-treated group.

In our previous studies, we discovered a pyrimidine derivative, benzyl 7-(4-hydroxy-3-methoxyphenyl)-5-methyl-4,7-dihydrotetrazolo[1,5-a] pyrimidine-6-carboxylate (BHDPC, ChemBridge ID: 7989205; the chemical structure is shown in **Figure 1A**) that can prevent chemical-induced cerebral hemorrhage injury in docking-based virtual screening and zebrafish models (Shen et al., 2013, 2015). In addition, we found that BHDPC could suppress 1-methyl-4-phenyl-1,2,3,6-tetrahydropyridine (MPTP)-induced dopaminergic neuron loss and locomotion deficiencies in a zebrafish model of Parkinson’s disease (PD) (Chong et al., 2015). Further studies of brain tissue slices and SH-SY5Y cells models demonstrated that BHDPC conferred neuroprotection via modulating intrinsic mitochondrial apoptotic pathways, which was closely associated with phosphorylation of PKA/CREB signaling (Chong et al., 2015). It is well known that microglial cell activation and sequential neuroinflammation play a critical role in neurodegenerative disorders. Our previous studies indicated a potential relationship between BHDPC and neuroinflammation. Therefore, in the current study, we aimed to investigate the anti-neuroinflammatory effects of BHDPC in LPS-stimulated BV-2 microglial cells *in vitro* and a LPS-induced mouse model *in vivo*. Moreover, the role of BHDPC in the NF-κB and PKA/CREB pathways was further investigated. Finally, the protective effects of BHDPC against neuroinflammation-induced neurotoxicity in HT22 mouse hippocampal cells co-cultured with BV-2 microglia were also explored.

## Materials and Methods

### Chemicals and Reagents

BHDPC was purchased from ChemBridge Corporation (ChemBridge ID: 7989205, San Diego, CA, United States). LPS (Escherichia coli serotype 055: B5) was purchased from Sigma-Aldrich (St. Louis, MO, United States). PKA-specific inhibitor (H-89) was purchased from Selleck Chemicals (Shanghai, China). All other chemicals and solvents were of molecular biology grade.

### Cell Culture and Treatment

BV-2 cells, previously characterized as a widely used immortalized murine microglial cell line (Blasi et al., 1990; Saleppico et al., 1996), were obtained from the Kunming Cell Bank of Type Culture Collection, Kunming Institute of Zoology. HT22 mouse hippocampal cells were obtained from the University of California. BV-2 cells and HT22 cells were cultured in RPMI 1640 and DMEM, respectively (Gibco, Carlsbad, CA, United States). The medium was supplemented with 10% FBS (Gibco), 100 U/ml penicillin (Gibco), and 100 μg/ml streptomycin. Cells were cultured in an atmosphere of 95% air and 5% CO_2_ at 37°C. LPS stock (10 μg/ml) and BHDPC stock (50 mM) were firstly prepared in PBS and DMSO, respectively, and then diluted into final doses.

### BV-2 Microglia and HT22 Mouse Hippocampal Cells Co-culture System

Neuroprotective effects of BHDPC were tested in a BV-2 microglia cell and HT22 mouse hippocampal cell co-culture system using Corning^®^ Transwell^®^ polycarbonate membrane cell inserts (12 mm Transwell with 0.4 μm pore size insert; Corning, New York, NY, United States). HT22 cells were cultured in a 24-well plate, and BV-2 cells were seeded on the Transwell insert that was then placed above the HT22 neuronal cells layer for co-culture. At 24 h after cell seeding, BV-2 cells were pretreated with various doses of BHDPC for 1 h and then co-stimulated with LPS (500 ng/ml) for another 24 h. After that, HT22 cells were then subjected to further assay.

### Cell Viability Assay

Cell viability was measured by the MTT assay. Briefly, cells were seeded in 24- or 96-well culture plates and received the indicated treatments. After that, cells were incubated with MTT and finally the absorbance at 570 nm was measured using a Flexstation 3 Microplate Reader (Molecular Devices, Sunnyvale, CA, United States).

### NO Assay and ROS Measurement

Microglial production of NO was assessed by measuring the accumulated nitrite released into culture media. Briefly, after the indicated treatment, culture media was collected and analyzed via a Nitric Oxide Colorimetric Assay kit according to the manufacturer’s protocol (BioVision, Milpitas, CA, United States). The level of ROS was analyzed using the fluorescent probe, CM-H_2_DCFDA (Molecular Probes, Eugene, OR, United States). Once treatment was finished, cells were collected, washed with PBS and incubated with the probe. After that, the ROS level was also measured using a Flexstation 3 Microplate Reader.

### Enzyme-Linked Immunosorbent Assay (ELISA) for TNF-α, IL-6, IL-1β, IL-10, and PGE_2_

The TNF-α, IL-6, IL-10, IL-1β, and PGE_2_ released in conditioned media were assessed by specific ELISA Ready-SET-Go kits (eBiosciences, San Diego, CA, United States). The levels were quantified following the manufacturer’s protocols.

### Mitochondrial Membrane Potential Measurement

After treatment, the cells were incubated with JC-1 (10 μg/mL) at 37°C for 15 min and then washed with PBS. For signal quantification, the intensity of red fluorescence and green fluorescence was determined. The ratio of JC-1 red/green fluorescence intensity was semi-quantitatively calculated and the value was normalized to the LPS-treated group.

### Cellular DNA Fragmentation Assay and Caspase 3 Activity Assay

The effect of BHDPC on activated microglial-induced cellular DNA fragmentation in HT22 cells was measured using a Cellular DNA Fragmentation ELISA kit (Roche Applied Science, Mannheim, Germany) according to the protocols. Absorbance at 450 nm was measured and represented as the level of DNA fragmentation. The activity of caspase 3 was measured using the commercially available EnzChek Caspase-3 Assay Kit (Invitrogen, San Diego, CA, United States) according to the manufacturer’s protocol.

### Electrophoretic Mobility Shift Assay (EMSA)

The nuclear protein was separated using the Nuclear and Cytoplasmic Protein Extraction Kit (P0028, Beyotime, Shanghai, China). The DNA binding activities of NF-κB was analyzed using an ELISA-based TransAM NF-κB p65 EMSA kit (ActiveMotif, Carlsbad, CA, United States) according to the manufacturer’s protocol. Briefly, binding buffer and nuclear extract (∼30 μg) were added to the assay plate. Then, the plate was incubated for 1 h at room temperature in the dark, followed by incubation with NF-κB p65 antibody (1:1000; 1 h). Absorbance at 450 nm was measured via microplate reader.

### Reporter Gene Luciferase Assays

Briefly, BV-2 cells were transfected using the pNFκB-luc reporter plasmid provided by the Cignal NFκB Reporter (luc) Kit (CCS-013L, SABiosciences, Frederick, MD, United States) using the Attractene Transfection Reagent (QIAGEN, Hilden, Germany) according to manufactrurer’s protocol. Transfection efficiency was controlled by co-transfection with Renilla luciferase reporter plasmid. About 24 h after transfection, the transfected cells received the indicated treatment. The samples were then subjected to the Dual-Luciferase^®^ Reporter Assay System (E1910; Promega, Madison, WI, United States), and luciferase activities were measured.

### Quantitative PCR (qPCR) Assay

Total RNA of BV-2 cells and brain samples was extracted using the High Pure RNA Isolation Kit (Roche Applied Science) according to the manufacturer’s protocol. Isolated RNA was then reverse-transcribed into cDNA using the Transcriptor First Strand cDNA Synthesis Kit (Roche Applied Science) following the standard protocol. The qPCR assay was conducted using FastStart Universal SYBR Green Master reagents (Roche Applied Science) with the Applied Biosystems 7900 HT Fast Real-Time PCR System (Applied Biosystems Inc., Foster City, CA, United States). The amplification parameters used here was 50°C for 2 min then 95°C for 10 min, followed by 40 cycles of 95°C for 15 s and 60°C for 30 s. Each sample was analyzed in triplicate and the relative expression of mRNA was calculated after normalization to GAPDH. The primer sequences used were also listed (**Table 1**).

**Table 1:** Primers used in qPCR assay.

Gene	Forward primer (5′→3′)	Reverse primer (5′→3′)
iNOS	CAAGAGTTTGACCAGAGGACC	TGGAACCACTCGTACTTGGGA
COX-2	TTGAAGACCAGGAGTACAGC	GGTACAGTTCCATGACATCG
TNF-α	CCTATGTCTCAGCCTCTTCT	CCTGGTATGAGATAGCAAAT
IL-1β	GGCAACTGTTCCTGAACTCAACTG	CCATTGAGGTGGAGAGCTTT
		CAGC
IL-6	CCACTTCACAAGTCGGAGGCTT	CCAGCTTATCTGTTAGGAGA
CD206	CTTCGGGCCTTTGGAATA AT	TAGAAGAGCCCTTGGGTTGA
Arg-1	GTGAAGAACCCACGGTCTGT	GCCAGAGATGCTTCCAACTG
YM1/2	CAGGGTAATGAGTGGGTTGG	CACGGCACCTCCTAAATTGT
GAPDH	ATGTACGTAGCCATCCAGGC	AGGAAGGAAGGCTGGAAGAG

### Immunohistochemistry

For BV-2 microglia cells staining, cells were washed, fixed by 3.7% PFA, permeabilized by 0.3% Triton X-100-PBS for 15 min, and then blocked for another 30 min at room temperature. After that, cells were incubated with rabbit anti-NF-kB p65 (1:500) overnight at 4°C and goat anti-rabbit, Alex Fluor 594 conjugate secondary antibody (111-585-003, 1:500, Jackson Immuno Research Laboratories, West Grove, PA, United States) for 1 h at room temperature in the dark. For the nuclei observation, the cells were further stained with DAPI solution (Thermo Scientific) for 10 min. Finally, the samples were mounted with the Prolong anti-fade reagent and imaged by the Leica TCS SP8 confocal laser scanning microscope system (Leica, Wetzlar, Germany).

### Preparation of Whole Cell, Cytoplasmic, and Nuclear Protein

For the whole cell protein extraction, BV-2 cells and mouse brain samples were washed with ice-cold PBS and incubated with RIPA lysis buffer (Beyotime). Cell lysates were centrifuged, and the supernatant was collected and stored. The subcellular fractionation was prepared using the Nuclear and Cytoplasmic Extraction Reagents (Thermo Scientific) according to the manufacturer’s f nuclear extraction kit protocol. The protein content was assayed using the BCA protein quantification kit (Thermo Scientific).

### Co-immunoprecipitation Assays

The immunoprecipitation was conducted using the Pierce^TM^ Protein A/G Magnetic Beads kit (Thermo Scientific) according to the protocols. In brief, cell protein extraction was performed and protein concentration quantified. Then, each protein extraction (∼500 μg) was mixed with immunoprecipitation antibody (CBP primary antibody, 5 μg). The mixture was incubated overnight at 4°C. After that, 25 μg of magnetic beads were added and incubated for 1 h at room temperature. Beads were then washed and antigen was eluted. For blots, 20 μl of target antigen in elution buffer was used and detected as described in the Western blot analysis.

### Western Blot

Aliquots protein samples (30∼40 μg) were resolved by SDS-PAGE (7.5–15%) and transferred to PVDF membranes. Membranes were blocked, followed by incubation at 4°C overnight with diluted primary antibodies: CBP (1:1000), p-IκBα (1:1000), IκBα (1:1000), p-IKKα/β (1:1000), IKKα (1:1500), p-PKA (1:1000), PKA (1:2000), p-CREB (1:1000), CREB (1:1500), p-NF-κB p65 (1:1000), NF-κB p65 (1:1000), β-actin (1:2000), β-Tubulin (1:2000), and GAPDH (1:2000) purchased from Cell Signaling Technology (Beverly, MA, United States); iNOS (1:500), COX-2 (1:1000), and IBA1 (1:1500) were purchased from Invitrogen. Membranes were washed and incubated with HRP-linked, anti-rabbit/mouse secondary antibody (1:2500, Cell Signaling Technology) for 1 h at room temperature. Finally, bands were visualized using an ECL plus Western Blotting Detection Reagents kit (GE Healthcare, Milwaukee, WI, United States). The membranes were then scanned on a Bio-Rad ChemiDoc XRS Imaging System, and the intensity of the protein bands was analyzed using Bio-Rad Quantity One Software (Bio-Rad, Hercules, CA, United States).

### Graphing and Statistical Analysis

Statistical analyses were performed using GraphPad Prism (ver. 6.0, GraphPad Inc., San Diego, CA, United States), and data were represented as the mean ± standard deviation (SD). The difference between two groups was compared by using an independent sample *t-test* and one- or two-way ANOVA with Bonferroni’s correction for multiple group comparison. Pearson’s correlation coefficient was used for the correlation analyses. *P <* 0.05 was considered significant.

## Results

### Effects of BHDPC on the Cell Viability of BV-2 Microglial Cells

In order to estimate the range of effective concentrations, we firstly detected the action of BHDPC on the cell viability of BV-2 cells. As shown in **Figure 1B**, subsequent determination indicated that BHDPC (0.3–30 μM) had no cytotoxic action on BV-2 cells with 24 h incubation (all *p* > 0.01, versus the control group). LPS (500 ng/mL) treatment for 24 h slightly decreased the cell viability to 96.12 ± 3.46%, with no significant difference compared to the control group (**Figure 1C**, *p* > 0.05, versus the control group). Furthermore, pretreatment with BHDPC (1–10 μM) was able to restore the decreased cell viability of BV-2 cells stimulated by LPS for 24 h (**Figure 1C**, *p* > 0.05, versus the LPS-treated group).

### BHDPC Suppressed the Expression of NO/iNOS and PGE_2_/COX-2, and the Production of Pro-inflammatory Cytokines in LPS-Activated BV-2 Microglial Cells

**FIGURE 2:**
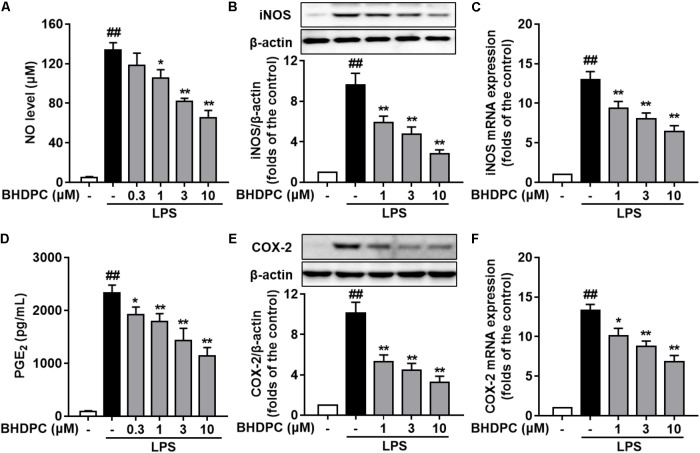
BHDPC blocked the NO/iNOS and PGE_2_/COX-2 pathways in LPS-activated BV-2 microglial cells. Cells were treated with BHDPC for 1 h, followed by LPS stimulation. The cytokine level was determined by Elisa. The mRNA and protein expression were determined by qPCR and Western blot assay, respectively. **(A,D)** BHDPC inhibited the production of NO **(A)** and PGE_2_ **(D)**. **(B,E)** BHDPC suppressed the expression of iNOS **(B)** and COX-2 **(E)**. **(C,F)** BHDPC decreased the mRNA expression of iNOS **(C)** and COX-2 **(F)**. The mRNA level was normalized to GAPDH and expressed as fold- increase. Data are presented as means ± SEM of three independent experiments in triplicate. Control group was untreated cells. ^#^*P <* 0.05 and ^##^*P <* 0.01, versus control group; ^∗^*P <* 0.05 and ^∗∗^*P <* 0.01, versus LPS-treated group.

We initially evaluated the effects of BHDPC on NO and PGE_2_ production and expression of iNOS and COX-2 in LPS-stimulated BV-2 cells. As illustrated in **Figures 2A,D**, production of NO and PGE_2_ was markedly increased by LPS (all *p* < 0.01, versus the control group). However, pretreatment with BHDPC (1, 3, and 10 μM) for 1 h markedly decreased LPS-induced NO and PGE_2_ increases in a concentration-dependent manner (**Figures 2A,D**, all *p* < 0.05, versus the LPS-treated group). Then, as shown in **Figures 2B,E**, LPS-induced expression of iNOS and COX-2 was significantly inhibited by BHDPC treatment (1, 3, and 10 μM) in a concentration-dependent manner (all *p* < 0.05 versus the LPS-treated group). The best inhibitory effect was found at the dose of 10 μM, evidenced by the highest inhibition of NO, iNOS, PGE_2_, and COX-2 production (all *p* < 0.05 versus the LPS-treated group). Correspondingly, pretreatment with 1, 3, and 10 μM BHDPC dose-dependently decreased the mRNA expression of iNOS and COX-2 in LPS-activated BV-2 microglia (**Figures 2C,F**, all *p* < 0.05 versus the LPS-treated group).

**FIGURE 3:**
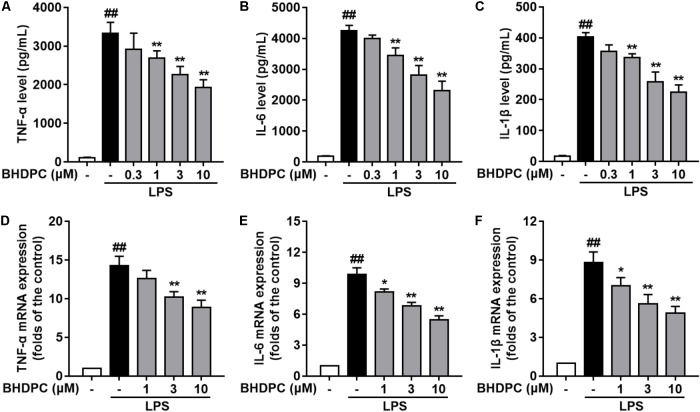
BHDPC suppressed the production of pro-inflammatory cytokines in LPS-activated BV-2 microglial cells. Cells were treated with BHDPC for 1 h, followed by LPS stimulation. The cytokine level was determined by Elisa. The mRNA and protein expression were determined by qPCR and Western blot assay, respectively. **(A–C)** BHDPC inhibited the production of TNF-α, IL-6, and IL-1β. **(D–F)** BHDPC decreased the mRNA expression of TNF-α, IL-6, and IL-1β. The mRNA level was normalized to GAPDH and expressed as fold- increase. Data are presented as means ± SEM of three independent experiments in triplicate. Control group was untreated cells. ^#^*P <* 0.05 and ^##^*P <* 0.01, versus control group; ^∗^*P <* 0.05 and ^∗∗^*P <* 0.01, versus LPS-treated group.

Consecutively, we investigated the effects of BHDPC on pro-inflammatory cytokines in LPS-activated BV-2 microglial cells. LPS stimulation significantly increased the production of TNF-α (**Figure 3A**), IL-6 (**Figure 3B**), and IL-1β (**Figure 3C**) in BV-2 cells, when compared to the control group (all *p* < 0.01, versus the control group). However, LPS-induced increased levels of TNF-α, IL-6, and IL-1β were significantly suppressed by pretreatment with BHDPC (1–10 μM) for 1 h in a dose-dependent fashion. Moreover, BHDPC (1–10 μM) also dose-dependently decreased the mRNA expression of TNF-α, IL-6, and IL-1β (**Figures 3D–F**, all *p* < 0.05 versus the LPS-treated group). Pretreatment with 10 μM BHDPC decreased the levels of TNF-α, IL-6, and IL-1β to 60.7%, 55.12%, and 54.5% of those of the LPS-treated group, respectively (all *p* < 0.01, versus the LPS-treated group).

### BHDPC Promoted Microglial Polarization Toward the M2 Anti-inflammatory Phenotype in LPS-Stimulated BV-2 Microglial Cells

**FIGURE 4:**
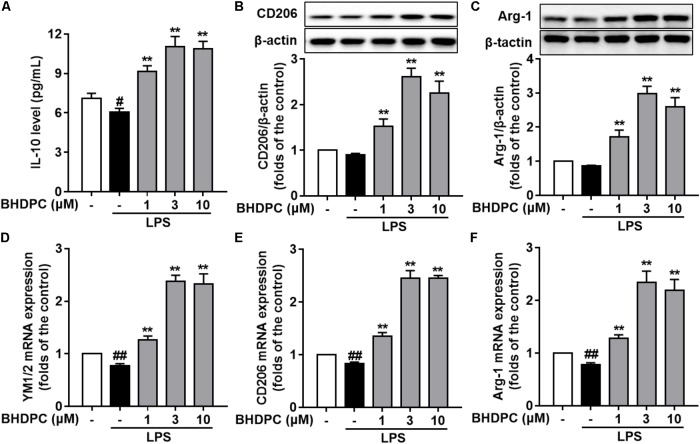
BHDPC promoted microglia polarization to the M2 phenotype in LPS-stimulated BV-2 microglial cells. Cells were treated with BHDPC for 1 h, followed by LPS stimulation. The cytokine level was determined by Elisa. The mRNA and protein expression were determined by qPCR and Western blot assay, respectively. **(A–C)** BHDPC increased M2 marker IL-10, CD206, and Arg-1 expression in BV-2 cells. **(D–F)** BHDPC prevented the LPS-induced down-regulation of YM1/2, CD206, and Arg-1 in mRNA level. The mRNA level was normalized to GAPDH and expressed as fold- increase. Data are presented as means ± SEM of three independent experiments in triplicate. Control group was untreated cells. ^#^*P <* 0.05 and ^##^*P <* 0.01, versus control group; ^∗^*P <* 0.05 and ^∗∗^*P <* 0.01, versus LPS-treated group.

Firstly, BHDPC (1, 3, and 10 μM) increased the expression of IL-10 (**Figure 4A**), CD206 (**Figure 4B**), and Arg-1(**Figure 4C**) in LPS-stimulated BV-2 cells (all *p* < 0.01 versus the LPS-treated group). Interestingly, we found that treatment with BHDPC at a dose of 3 μM had the best effect on M2 marker induction, which increased the level of IL-10, CD206, and Arg-1 to ∼1.6, ∼2.6, and ∼2.9-fold that of the control group, respectively (all *p* < 0.01). In addition, LPS stimulation decreased mRNA expression of YM1/2, CD206, and Arg-1(**Figures 4D–F**), whereas BHDPC incubation significantly reversed this tendency. As expected, 3 μM BHDPC had the best potency, and increased the level of YM1/2, CD206 and Arg-1 to ∼2.3, ∼2.4, and ∼2.5-fold that of the control group in LPS-stimulated BV-2 microglial cells (both *p <* 0.01).

### BHDPC Prevented LPS-Activated BV-2 Microglia-Mediated Neurotoxicity in HT22 Hippocampal Cells

**FIGURE 5:**
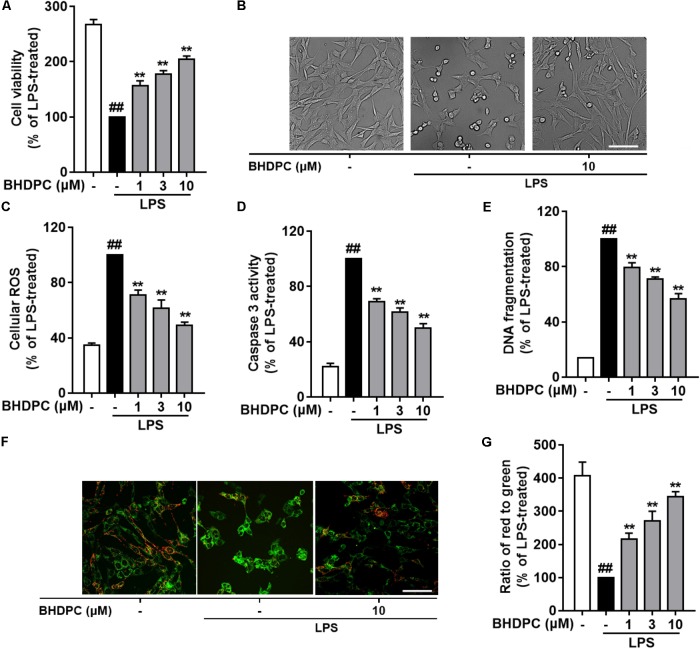
BHDPC provided neuroprotective effects against the HT22 cells damages induced by LPS-activated BV-2 microglia. BV-2 cells were co-cultured on Transwell inserts with HT22 cells. Microglial were pretreated with BHDPC for 1 h, and then stimulated with LPS. **(A)** The cell viability was measured via MTT assay. **(B)** Cellular morphology of HT22 cells. **(C,D)** The cellular ROS level and caspase 3 activity were measured via the fluorogenic probe and Caspase-3 Assay Kit, respectively. **(E)** Cell DNA fragmentation was measured via ELISA assay. **(F,G)** The mitochondrial membrane potential (MMP) was detected using JC-1 dye. Data are presented as means ± SEM of three independent experiments in triplicate. Control group was untreated cells. ^#^*P <* 0.05 and ^##^*P <* 0.01, versus control group; ^∗^*P <* 0.05 and ^∗∗^*P <* 0.01, versus LPS-treated group.

Since BHDPC showed inhibitory effects on neuroinflammation in LPS-activated microglia, we then investigated whether BHDPC could prevent neuroinflammation-mediated neuronal death using the BV-2 microglia and HT22 cells co-culture system. As shown in **Figures 5A,B**, LPS stimulation induced a significant reduction in the cell viability of co-cultured HT22 cells (*p* < 0.01, versus the control group). Pre-treatment with BHDPC (1, 3, and 10 μM) significantly prevented HT22 cells against activated microglia-induced cytotoxicity, and increased the cell viability to ∼156.2, ∼177.5, and ∼204.3% of that of the LPS-treated group, respectively, in a dose-dependent manner (all *p* < 0.01). Moreover, we also found that LPS stimulation significantly increased intracellular ROS production in HT22 neurons (**Figure 5C**). However, the LPS-induced ROS production was blocked by BHDPC (1, 3, and 10 μM) treatment in a dose-dependent fashion (*p* < 0.01, versus the LPS-treated group). As shown in **Figure 5D**, further results on HT22 cells caspase 3 activity also showed that LPS stimulation significantly increased caspase 3 activity to ∼ 454.3% that of the control group, whereas BHDPC (1, 3, and 10 μM) incubation resulted in significant reduction of caspase 3 activity, in a dose-dependent manner (all *p* < 0.01). In addition, the results of DNA fragmentation also indicated that BHDPC (1, 3, and 10 μM) incubation could protect against cell damage, as evidenced by significant reductions in DNA fragmentation to ∼79.2, ∼70.1, and ∼56.4% that of the LPS-treated group, respectively (**Figure 5E**, all *p* < 0.01). In addition, JC-1 dye was used to monitor the mitochondrial membrane potential (MMP); as illustrated in **Figures 5F,G**, LPS stimulation induced significant MMP dissipation. However, BHDPC at 1, 3, and 10 μM attenuated LPS-activated BV-2-induced MMP loss in a concentration-dependent manner (all *p* < 0.01).

### BHDPC Inhibited the Activation of NF-κB Pathway in LPS-Activated BV-2 Microglial Cells and LPS-Challenged Mouse Brains

**FIGURE 6:**
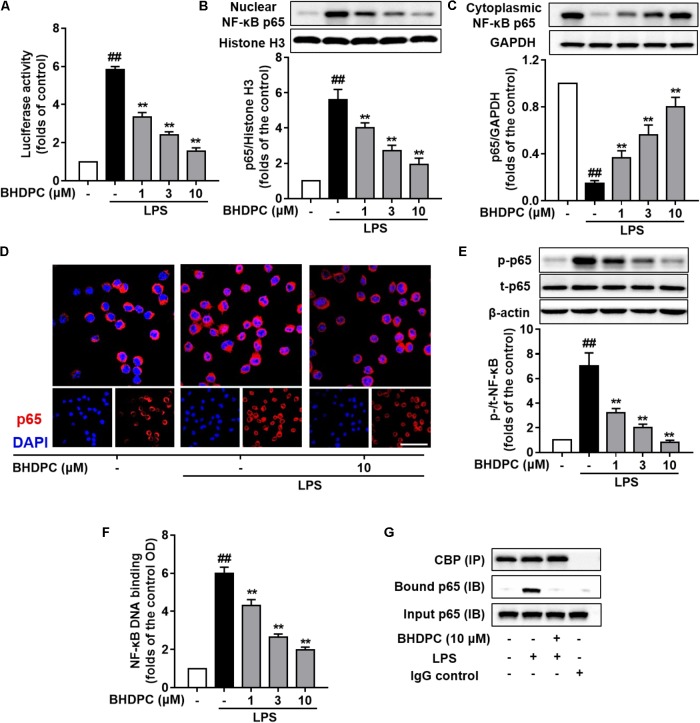
BHDPC inhibited NF-κB activation in LPS-stimulated BV-2 microglial cells. **(A)** BHDPC suppressed the NF-κB reporter luciferase activity in LPS-activated BV-2 cells. Transfected cells were pretreated with BHDPC for 1 h, and then stimulated with/without LPS. The luciferase activity was measured and then expressed as fold-increase. **(B–D)** BHDPC suppressed NF-κB p65 translocation in LPS-activated BV-2 cells. The whole cell, cytoplasmic, and nuclear protein were prepared and the levels of cytoplasmic p65 **(B)** and nuclear p65 **(C)** were measured using Western Blot. **(D)** The NF-κB p65 protein localization was also detected via immunofluorescence staining. The NF-κB p65 proteins were labeled by p65 primary antibody and Alexa Fluor 594-conjugated secondary antibody. The nucleus was counterstained with DAPI. **(E)** The level of phosphorylation p65 phosphorylation were measured using Western Blot. **(F)** StA inhibited DNA binding of NF-κB in LPS-stimulated BV-2 microglia. Nuclear extracts were prepared and subjected to the ELISA-based DNA binding assay. The absorbance was measured using a microplate reader. **(G)** BHDPC inhibited LPS-induced formulation of NF-κB and coactivator CBP complex, as detected via the co-immunoprecipitation. Data are presented as means ± SEM of three independent experiments in triplicate. Control group was untreated cells. ^#^*P <* 0.05 and ^##^*P <* 0.01, versus control group; ^∗^*P <* 0.05 and ^∗∗^*P <* 0.01, versus LPS-treated group.

To explore the role of BHDPC in NF-κB activation, the NF-κB luciferase reporter system was used to determine NFκB-regulated gene transcription and signal transduction. Results indicated that BHDPC (1, 3, and 10 μM) could inhibit the NF-κB-driven gene transcriptional activity increased by LPS stimulation in a dose-dependent fashion (**Figure 6A**, all *p* < 0.01 versus LPS-treated group).

Next, we examined the effects of BHDPC on nuclear translocation of the NF-κB p65 subunit. As shown in **Figure 6B**, BHDPC (1, 3, and 10 μM) significantly blocked the NF-κB p65 LPS-activated nuclear translocation (**Figure 6C**, *p* < 0.01). The nuclear translocation was further revealed by immunofluorescence assay. As expected, BHDPC (10 μM) pretreatment significantly reduced the LPS-stimulated accumulation of NF-κB p65 in the nuclei (**Figure 6D**). Further, we found that BHDPC significantly reduced the NF-κB p65 phosphorylation induced by LPS (**Figure 6E**, all *p* < 0.01). Corroboratively, the ELISA-based EMSA was conducted to assess the binding of NF-κB to DNA, and the results showed that BHDPC (1, 3, and 10 μM) significantly abolished the LPS-induced NF-κB-DNA binding activity in a dose-dependent manner (**Figure 6F**, *p* < 0.01). Moreover, the co-immunoprecipitation results indicated that the formation of the NF-κB and coactivator CBP complex detected in LPS-induced BV-2 cells (**Figure 6G**) was reversed by BHDPC treatment.

**FIGURE 7:**
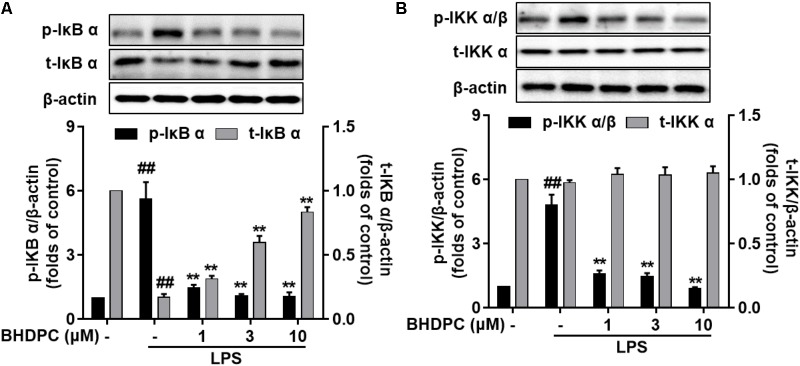
BHDPC inhibited LPS-induced IKK α/β phosphorylation, IκB α phosphorylation, and IκB α degradation in activated BV-2 microglial cells. Cells were treated with BHDPC for 1 h, followed by LPS stimulation. After incubation, protein was prepared and the levels of **(A)** total/phosphorylated IκB α and total/phosphorylated IKK α/β **(B)** were measured via Western Blot. Data are presented as the mean ± SD of three independent experiments (*n* = 9). Data are presented as means ± SEM of three independent experiments in triplicate. Control group was untreated cells. ^#^*P <* 0.05 and ^##^*P <* 0.01, versus control group; ^∗^*P <* 0.05 and ^∗∗^*P <* 0.01, versus LPS-treated group.

In LPS-activated BV-2 microglial cells, we found that the levels of phosphorylated IKKα/β and IκBα were markedly increased (**Figures 7A,B** *p* < 0.01 versus the control). However, BHDPC significantly attenuated the increase in phosphorylation of IKKα/β and IκBα in a dose-dependent manner. Notably, 10 μM BHDPC reduced the levels of phosphorylated IKKα/β and IκBα to ∼18.7%, and 17.7% of those of the LPS-treated group, respectively. In addition, LPS decreased IκBα degradation in BV-2 cells, which was reversed by BHDPC (1, 3, and 10 μM) (**Figure 7B**, *p* < 0.01).

### BHDPC Promoted PKA/CREB Phosphorylation in LPS-Activated BV-2 Microglial Cells and LPS-Challenged Mice Brains

**FIGURE 8:**
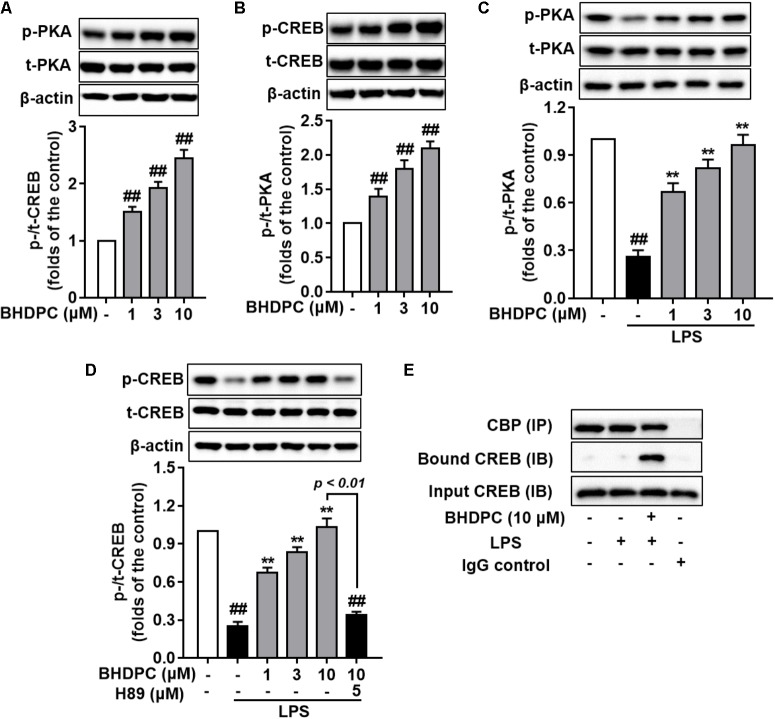
BHDPC induced the phosphorylation of PKA and CREB and formation of CREB-CBP complex in BV-2 microglial cells. Cells were firstly incubated with H89 for 30 min and treated with BHDPC for 1 h, after that treated with or without LPS. **(A,B)** BHDPC induced phosphorylation of PKA and CREB in BV-2 cells without LPS stimulation. **(C,D)** BHDPC promoted phosphorylation of PKA and CREB in LPS-activated BV-2 microglial cells. **(E)** BHDPC induced the formation of CREB and coactivator CBP complex detected via the co-immunoprecipitation. Data are presented as means ± SEM of three independent experiments in triplicate. Control group was untreated cells. ^#^*P <* 0.05 and ^##^*P <* 0.01, versus control group; ^∗^*P <* 0.05 and ^∗∗^*P <* 0.01, versus LPS-treated group.

Encouraged by the elevated phosphorylation of PKA/CREB mediated by BHDPC in SH-SY5Y cell lines in our previous study, here we further assessed the intracellular cAMP level and phosphorylation of PKA and CREB in BV-2 microglial cells. Firstly, we found that BHDPC dose-dependently promoted CREB and PKA phosphorylation (**Figures 8A,B**, all *p* < 0.01 versus the control group) in BV-2 microglia cell without LPS stimulation. Further results also indicated that BHDPC (1, 3, and 10 μM) significantly reversed the LPS-induced reduction of PKA phosphorylation (**Figure 8C**, all *p* < 0.01). In addition, BHDPC significantly reversed the decrease of CREB phosphorylation induced by LPS in a dose-dependent fashion (**Figure 8D**, all *p* < 0.01 versus the LPS-treated group), whereas this effect was abolished by pretreatment with the PKA inhibitor, H89. Furthermore, co-immunoprecipitation of CREB with CBP from BHDPC-treated BV-2 cells indicated that BHDPC promoted formulation of the CREB-CBP complex (**Figure 8E**).

### Blocking the PKA Pathway Reversed BHDPC-Mediated Anti- neuroinflammatory and Neuroprotective Actions

**FIGURE 9:**
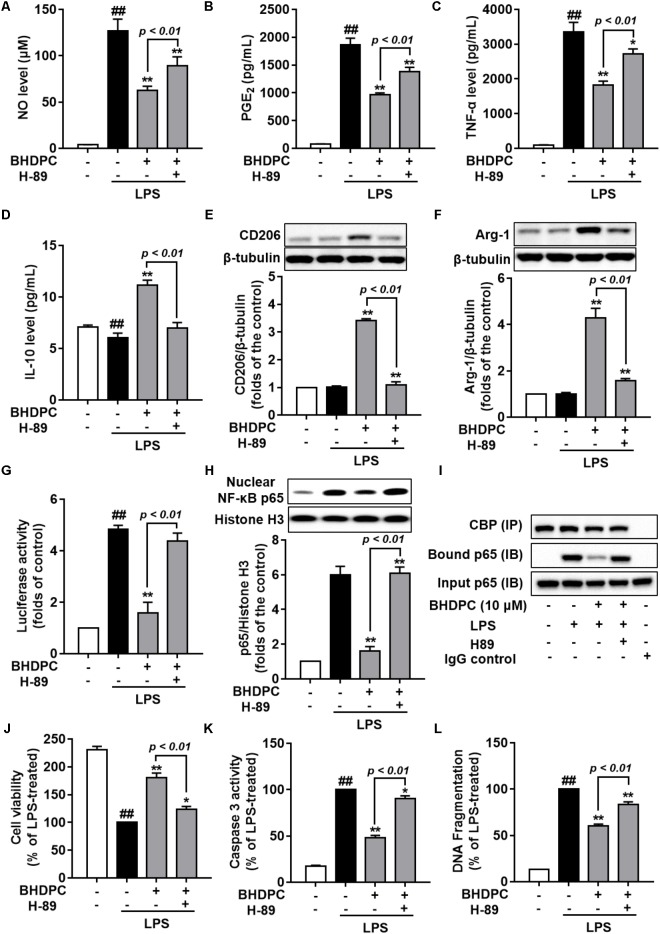
Blocking the PKA pathway reversed BHDPC-mediated anti-neuroinflammatory and neuroprotective actions. BV-2 cells were pretreated with 10 μM BHDPC or 5 μM H89 for 1 h, and then exposed to LPS. PKA inhibitor H89 reversed BHDPC-mediated inhibition of NO **(A)**, PGE_2_ **(B)**, and TNF-α expression **(C)**. **(D–F)** PKA inhibition abrogated BHDPC-induced expression of IL-10 **(D)**, CD206 **(E)**, and Arg-1 **(F)**. PKA inhibitor reversed the inhibitory actions of BHDPC on NF-κB reporter luciferase activity **(G)**, p65 translocation **(H)**, and formulation of NF-κB and CBP complex **(I)**. Cells were pretreated with BHDPC or H89 for 1 h, and then exposed to LPS for 24 h. PKA inhibitor reversed the protective actions of BHDPC on cell viability **(J)**, cell caspase-3 activity **(K)**, and DNA fragmentation **(L)**. Data are presented as means ± SEM of three independent experiments in triplicate. Control group was untreated cells. ^#^*P <* 0.05 and ^##^ *P <* 0.01, versus control group; ^∗^*P <* 0.05 and ^∗∗^*P <* 0.01, versus LPS-treated group.

We next used the PKA inhibitor, H89 to block the PKA activity in BV-2 microglial cells, to test whether BHDPC-mediated inhibitory effects against neuroinflammation were mediated by PKA. We found that H89 significantly abolished the BHDPC-mediated inhibition of production of pro-inflammatory mediators and cytokines, including NO (**Figure 9A**), PEG2 (**Figure 9B**), and TNF-α (**Figure 9C**) (all *p* < 0.01). Furthermore, we found that blocking the PKA activity also abrogated the effect of BHDPC on promoting M2 marker protein expression, as evidenced by the levels of IL-10, CD206, and Arg-1 being decreased when compared to that of the group only incubated with BHDPC but not H89 (**Figures 9D–F**). Interestingly, we also found that PKA inhibitor suppressed the inhibitory effects of BHDPC on NF-κB activation. H89 aborted the inhibitory effect of BHDPC on NF-κB-driven gene transcriptional activity (**Figure 9G**) and p65 nucleus translocation (**Figure 9H**). Also, the suppressive action on the formation of the NF-κB and CBP complex was abolished by PKA inhibitor (**Figure 9I**). Moreover, PKA inhibitor H89 also abolished the BHDPC-mediated neuroprotective actions in HT22 cells. As shown in **Figure 9J**, H89 treatment reversed the BHDPC-mediated increase of cell viability of HT22 cells in the co-culture system (*p* < 0.01). Moreover, PKA inhibition by H89 also abrogated BHDPC-mediated inhibition of caspase 3 activity of HT22 cells in the co-culture system (**Figure 9K**, *p* < 0.01). Further results on the DNA fragmentation of HT22 cells also showed that PKA inhibitor abolished the protective effects of BHDPC against LPS-activated BV-2 microglia-induced HT22 cell damage, as evidenced by the significant increase in DNA fragmentation compared to the group without H89 incubation (**Figure 9L**, *p* < 0.01).

## Discussion

Neuroinflammatory responses are inevitable and important pathological processes in several kinds of CNS disorder (Xanthos and Sandkuhler, 2014; Mietto et al., 2015). Microglia are immune-surveillance cells and serve as major components of inflammation. (Xanthos and Sandkuhler, 2014; Chuang et al., 2015; Mietto et al., 2015). Therefore, alleviating activated microglia-induced inflammatory processes and subsequent cellular damage might be a valuable therapeutic approach for neuroinflammation-related diseases (Gemma, 2010; Dhama et al., 2015). In this study, we showed for the first time that the novel neuroprotectant, BHDPC could inhibit neuroinflammatory responses in LPS-activated BV-2 microglia cells. Moreover, BHDPC provided protection against neuroinflammation-induced neurotoxicity in HT22 mouse hippocampal cells. Furthermore, the results demonstrated that the effects of BHDPC were mediated by the inactivation of NF-κB and activation of PKA/CREB signaling.

It is well known that the NO/iNOS and PGE_2_/COX-2 signal pathways are significantly up-regulated in LPS-activated BV-2 microglial cells. iNOS and COX-2 are associated with neuronal inflammation and subsequent pathogenesis of CNS diseases (Minghetti and Levi, 1998). Therefore, agents that could regulate levels of iNOS and COX-2 might be able to prevent and treat neuroinflammation-related disorders (David and Kroner, 2011; Saijo and Glass, 2011; Joseph and Venero, 2013; Mantovani et al., 2013; Kwon et al., 2017). In this study, we firstly found that BHDPC could suppress both NO/iNOS and PGE_2_/COX-2 signaling pathways in LPS-activated BV-2 microglial cells. Next, we found that BHDPC could also decrease the expression of pro-inflammatory cytokines. These pro-inflammatory cytokines could promote neuronal damage, and also induce more microglia activation as a feedback mechanism (Block et al., 2007). Therefore, modulating cytokine function is one mechanisms by which to control inflammation-induced CNS disorders (Minghetti and Levi, 1998; Block et al., 2007). Hence, the current data demonstrated that BHDPC was able to attenuate aberrant microglial activation and activated the microglia-induced inflammatory response.

Similar to macrophages, microglial cells also show plasticity, and polarize into different functional phenotypes. In response to various microenvironmental disturbances, microglia can be phenotypically polarized into classical (pro-inflammatory; M1) and alternative (anti-inflammatory; M2) phenotypes (Lu et al., 2010; Zhou et al., 2014b). It is well known that the anti-inflammatory M2 phenotype confers neuroprotective effects via enhancing the expression of cytokines and proteins involved in inflammatory resolution, homeostasis, and wound-healing (David and Kroner, 2011; Saijo and Glass, 2011; Joseph and Venero, 2013; Mantovani et al., 2013). Inhibiting the M1 phenotype while stimulating the M2 phenotype has been suggested as a more viable potential strategy for the treatment of neuroinflammatory disorders (Hu et al., 2015). For instance, two agents approved for multiple sclerosis (Freigang et al., 2011) therapy, glatiramer acetate and β-interferon, have demonstrated not only M1-inhibiting actions, but also promote a balance between M1 and M2 cells (Burger et al., 2009; Kieseier, 2011). Our current results indicated that BHDPC could significantly promote microglial polarization toward the anti-inflammatory M2 phenotype in LPS-stimulated BV-2 microglial cells. Taken together, results suggest that BHDPC could promote microglial M2 phenotype polarization, which might contribute to BHDPC-induced neuroinflammation.

Microglia-mediated neuroinflammation is characterized by pro-inflammatory molecule production, these molecules are neurotoxic and subsequently result in death and damage of neighboring neurons (Chan et al., 2017; Chen et al., 2017; Alcalde-Estevez et al., 2018). Therefore, agents that are able to block pro-inflammatory mediators would be expected to confer neuroprotection. The neuron-microglia co-culture system provides an environment for both microglia and neurons to grow together in close proximity; hence, it is widely used to test the neuroprotective effects of anti-inflammatory compounds. In this study, we also used HT22 hippocampal neurons and the BV-2 microglia co-culture system to further explore BHDPC-mediated actions against inflammation-induced neurotoxicity. The BV-2 microglia were cultured in a transwell system that was placed above the HT-22 neuronal layer. The conditioned media from LPS-stimulated microglia was diffused into the HT-22 neuronal cells, resulting the HT-22 neurons death and apoptosis. Firstly, we confirmed that BHDPC could significantly alleviate LPS-stimulated microglia-induced cell toxicity in HT-22 cells. Furthermore, BHDPC was able to block the excess intracellular ROS level of HT-22 stimulated by activated microglia. Intracellular ROS served as an important second messenger that can activate a cascade of harmful events resulting in neuronal cell death and damage (Zhou et al., 2014a; Jiang et al., 2015). It is well known that loss of MMP is a hallmark of early-stage apoptosis and caspase 3 activation plays a key role in the execution phase of apoptosis. In this study, we also found that BHDPC could supress not only MMP loss, but also caspase 3 activation in co-culture HT-22 cells. These observations were further confirmed in experiments showing that BHDPC prevented inflammation-induced DNA fragmentation in HT22 cells. Taknen together, the current results clearly demonstrate the inhibitory actions of BHDPC against neuroinflammation-induced cell apoptosis.

The NF-κB signaling pathway is well known to be a central regulator of several kinds of inflammatory responses, including microglial-mediated neuroinflammation (Baeuerle and Henkel, 1994; Karin and Ben-Neriah, 2000). Since we found that BHDPC could abate pro-inflammatory responses, investigation of the NF-κB pathway might help us to further explain the underlying mechanisms. Firstly, the inhibitory activity of BHDPC against NF-κB transduction was confirmed in this study (Iu et al., 2017; Khan et al., 2017). Next, BHDPC-induced suppression of NF-κB p65 phosphorylation was also found in LPS-stimulated BV-2 microglia. Furthermore, BHDPC inhibited nuclear translocation of NF-κB in LPS-treated BV-2 microglia. Moreover, BHDPC abated NF-κB-DNA binding activity and the interaction of NF-κB with CBP. All of these results demonstrated that BHDPC could suppress NF-κB activation in BV-2 cells. Furthermore, we then tested the action of BHDPC in regulating IKK and IκB, two important upstream modulators of NF-κB signal pathways (Baeuerle and Henkel, 1994; Karin and Ben-Neriah, 2000; Baima et al., 2010). We found that BHDPC could block not only phosphorylation of IKK and IκB, but also degradation of IκB in LPS-activated microglia. Taken together, these data sugges that BHDPC could inhibit NF-κB activation, which might be associated with BHDPC-mediated anti-neuroinflammatory actions.

In our previous study, we found that BHDPC could activate PKA/CREB signaling in neuroblastoma SH-SY5Y cells. Similarly, in this study, we also found that BHDPC obviously induced the phosphorylation of PKA and CREB in both microglial cells and mouse brain. Notably, the BHDPC-induced phosphorylation of CREB was abolished by PKA inhibitor H89 in BV-2 microglia, which further indicated that the BHDPC-mediated CREB activation was also dependent on PKA activation. Once CREB becomes phosphorylated at Ser133, it can interact with CBP and induce the expression of anti-inflammatory cytokine genes, among which IL-10, IL-4, and IL-13 are the most important for reducing inflammation. Here, we also demonstrated that BHDPC promoted the CREB-CBP complex in LPS-stimulated microglial cells. These results were consistent with previous findings showing that BHDPC increased the expression of IL-10 and other M2 markers in LPS-activated microglia. Moreover, promoting the formation of the CREB-CBP complex could also result in the displacement of NF-κB from CBP and block NF-κB-CBP complex formulation, which inhibited expression of a variety of pro-inflammatory genes whose transcriptions are positively regulated by the NF-κB signaling pathway (Medzhitov and Horng, 2009; Ghosh et al., 2016; Park et al., 2016). Encouraged by the effect of BHDPC on PKA/CREB phosphorylation, we next assessed the role of PKA activation by BHDPC in LPS-induced neuroinflammation. Firstly, we observed that PKA inhibition using PKA inhibitor H89 obviously abolished the inhibitory effects of BHDPC on the production of inflammatory mediators and cytokines, including NO, PGE_2_, and TNF-α in LPS-stimulated BV-2 microglia cells. Moreover, BHDPC-mediated promotion of microglial M2 phenotype polarization was also blocked by PKA inhibitor. Interestingly, the BHDPC-induced NF-κB inactivation was also abrogated in condition of PKA inhibiton. Previous studies also indicacted that PKA activation could supress the activation of NF-κB in microglia, but PKA inhibitor or gene slicnece could revised PKA activator-mediated NF-κB incativation. Our results indicated that BHDPC might be a potent PKA activator, but further details need to be exploredin the following study. Furthermore, we also demonstrated PKA inhibition by a specific inhibitor also abolished the inhibitory actions of BHDPC against neuroinflammation-induced cell death and apoptosis. Therefore, we suggest that PKA-dependent CREB phosphorylation induced by BHDPC contributes to BHDPC-mediated anti-inflammation and neuroprotection.

In summary, our study demonstrated several principal findings, as follows: (1) BHDPC could inhibit neuroinflammatory response in LPS activated BV-2 microglia cells; (2) BHDPC provided protective actions against neuroinflammation-induced neurotoxicity in hippocampal cells; (3) BHDPC conferred anti-inflammatory and neuroprotective effects via the inactivation of NF-κB and activation of PKA/CREB signaling.

## Ethics Statement

All animal experiments were performed according to the National Institutes of Health Guide for the Care and Use of Laboratory Animals. All animal experiments were performed with prior approval from the Institutional Animal Ethics Committee (UMARE-007-2016 and UMARE-AMEND-042). Anesthesia will be done when there is an invasive operation or before sacrifice. The Animal sacrifice was conducted via the animal anesthesia system using carbon dioxide/oxygen gas. Enough food and clean water were ensured. Enough space was given to the animal to avoid over-crowding. During animal handling, the procedure should be mild and quiet to prevent over-reaction and harm to the animals.

## Author Contributions

MH, YZ, and SL conceived and designed the study. CL, TC, HZ, and YF performed the experiments. CL, DM, CZ, and SL drafted the manuscript.

## Conflict of Interest Statement

The authors declare that the research was conducted in the absence of any commercial or financial relationships that could be construed as a potential conflict of interest.

## Abbreviations

CNS: central nervous system
COX-2: cyclooxygenase-2
CREB: cAMP-response element-binding protein
IL: interleukin
iNOS: inducible nitric oxide synthase
LPS: lipopolysaccharide
MAPK: mitogen-activated protein kinase
MTT: 3-(4,5-dimethylthiazol-2-yl)-2,5-diphenyltetrazolium bromide
NF-κB: nuclear factor kappa B
NO: nitric oxide
PKA: cAMP-dependent protein kinase A
TNF: tumor necrosis factor.
